# mHealth Interventions in Low and Middle-Income Countries: A Systematic Review

**DOI:** 10.5539/gjhs.v8n9p183

**Published:** 2015-01-22

**Authors:** Kathryn Hurt, Rebekah J. Walker, Jennifer A. Campbell, Leonard E. Egede

**Affiliations:** 1 Center for Health Disparities Research, Medical University of South Carolina, Charleston, SC, United States; 2 Health Equity and Rural Outreach Innovation Center, Charleston VA COIN, Ralph H. Johnson VA Medical Center, Charleston, SC, United States; 3 Division of General Internal Medicine and Geriatrics, Department of Medicine, Medical University of South Carolina, Charleston, SC, United States

**Keywords:** mHealth, low-income countries, middle-income countries, developing countries

## Abstract

The purpose of this review was to determine whether mHealth interventions were effective in low- and middle-income countries in order to create a baseline for the evidence to support mHealth in developing countries. Studies were identified by searching Medline on 02 October 2014 for articles published in the English language between January 2000 and September 2014. Inclusion criteria were: 1) written in English, 2) completion of an mHealth intervention in a low or middle-income country, 3) measurement of patient outcomes, and 4) participants 18 years of age or older. 7,920 titles were reviewed and 7 were determined eligible based on inclusion criteria. Interventions included a cluster randomized trial, mixed methods study, retrospective comparison of an opt-in text message program, a two-arm proof of concept, single arm trial, a randomized trial, and a single subject design. Five out of seven of the studies showed significant difference between the control and intervention. Currently there is little evidence on mHealth interventions in developing countries, and existing studies are very diverse; however initial studies show changes in clinical outcomes, adherence, and health communication, including improved communication with providers, decrease in travel time, ability to receive expert advice, changes in clinical outcomes, and new forms of cost-effective education. While this initial review is promising, more evidence is needed to support and direct system-level resource investment.

## 1. Introduction

Chronic diseases are the most frequent cause of death and disability globally (Viswanathan et al., 2012). The burden of chronic diseases is progressing due to economic and social changes, growing populations, and scientific and industrial breakthroughs (Skolnik, 2012). Currently, the leading cause of death in low and middle income countries is non-communicable diseases, accounting for about 54% of all deaths (Skolnik, 2012). In addition to medical burden, costs of health care are an added burden of chronic disease, especially to people living at a low socioeconomic status (Skolnik, 2012). Out-of-pocket expenditures can impact financial status and further push individuals into poverty (Skolnik, 2012). In addition, malnutrition due to illness can affect quality of life and limit schooling (Skolnik, 2012). An aspect of many chronic diseases is self-care management and/or medication adherence in order to improve quality of life, health outcomes, and cost-effective healthcare (Hamine et al., 2015). Typically, only 50% of patients diagnosed with chronic diseases maintain chronic disease management regimes and the extent of non-compliance is even higher in developing countries (Hamine et al., 2015).

Based on popularity, availability, portability, and technological capacity, mobile phones and mHealth have a huge potential to impact chronic disease management by offering a way to increase access to healthcare (Hamine, 2015). Access to health-care is a significant factor in achieving both the Millennium Development Goals (MDGs) and the post-2015 Sustainable Development Goals (Royston et al., 2015). The need to improve the obtainability and use of healthcare information is also compelling, being highlighted in three of the MDGs that particularly addressed health: reducing child mortality, eradicating HIV/AIDS, and improving maternal health (Hagar et al., 2015). Having access to health information is vital, specifically for individuals without regular access to trained professionals to teach them how to properly care for themselves (United Nations, 2014). The implementation of mobile phone information systems could provide cost effective delivery strategies for healthcare, provide new ways to interact with the provider, and assist with travel and adherence (Tomlinson, 2013).

Opportunities to have health information on phones and/or via internet access has grown significantly (Royston, 2015). According to the International Telecommunication Union, there are now nearly 5 billion mobile phone subscriptions worldwide, with more than 85% of the global population having access to a commercial wireless signal (National Institute of Health [NIH], 2014). This saturation of mobile phone networks in various low and middle income countries has even been found to exceed the development of roads and electricity (NIH, 2014). And, in many of the developing countries, access to mobile phones is much easier than access to a regular doctor visit. (NIH, 2014) For example, 52.4% of the population are mobile subscribers in Nigeria, 58.4% in Kenya, and 57.9% in India (Royston et. al, 2015). Mobile phone use has been accepted across all demographics and socioeconomic groups and found to appear more in populations that are in need of health interventions (World Health Organization [WHO], 2011). As a result of increased technological innovations, the mHealth field is vastly growing, but the use of mHealth often remains untested (Tomlinson et al., 2013). Recent studies show that mHealth is a vital emerging technology to assist in self-care activities for patients, which could include text messaging or mHealth applications (apps) (Humble et al., 2015). More studies are needed using mHealth technology to provide a stronger based evidence for mHealth technology (Tomlinson et al., 2013).

To create a baseline for the evidence to support mHealth in developing countries, we conducted a literature review of interventions that used mHealth and measured outcomes. The goal of this systematic review was to determine whether outcomes of mHealth interventions have been reported from low and middle income countries, and if they were effective. For the purpose of this review, we used the definition of mHealth given by the World Health Organization, “mHealth is a part of eHealth, and concerns the use of mobile phones and related wireless devices by individuals, families, patients, carriers, and healthcare professionals to obtain or provide health services and information on health and healthcare” (Royston et al., 2015).

## 2. Materials and Methods

A systematic approach was taken to identify peer-reviewed articles where an mHealth intervention was completed in low and middle income countries. Studies were identified by searching Medline on 02 October 2014 for articles published in the English language between January 2000 and September 2014. The search strategy is described in Box 1.

**Box 1: Search strategy**

**Global Health**

- Text word ‘global health’
- Text word ‘international health’
- Text word ‘public health’

**Low Resource Country**

- Text word ‘low income’
- Text word ‘middle income’
- Text word ‘developing country’
- Text word ‘low resource’

**Intervention**

- Text word ‘intervention’
- Text word ‘effectiveness’
- Text word ‘evaluation’
- Text word ‘trial’
- Exploded MeSH ‘intervention studies’

**Papers Used**

- Any in ‘global health’ category and any in ‘low resource country’ category and any in ‘intervention’ category

### 2.1 Study Selection and Data Collection

**Figure 1:**
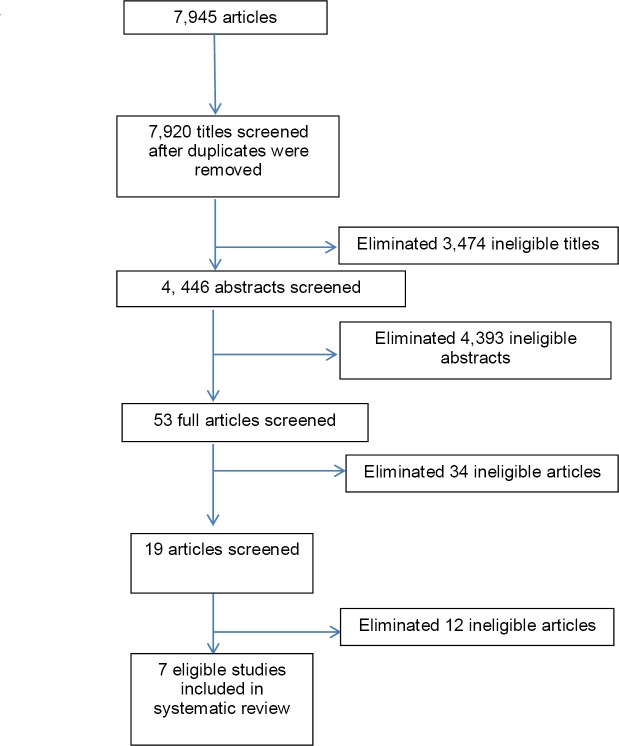
Search strategy

Eligibility assessment was performed in a standardized manner and is shown in Figure 1. Inclusion criteria were: 1) written in English, 2) completion of an mHealth intervention in a low or middle income country, 3) measurement of patient outcomes, and 4) participants 18 years of age or older. Three independent authors reviewed articles meeting inclusion criteria. Titles and abstracts were evaluated by using a standardized checklist. Abstracts were eliminated if they did not meet the inclusion criteria. Interventions included randomized controlled trials (RCTs) and quasi-experimental studies with or without a control arm.

For each study, data was obtained on the number of participants, sample population, intervention duration, mHealth delivery system, study design, major findings, and limitations. An outcome table for the intervention results was created to include the intervention description, intervention outcomes, major findings, and limitations. Interventions were too heterogeneous to allow a meta-analysis.

## 3. Results

### 3.1 Study Selection

A total of 7,945 papers were retrieved. After removing duplicates 7,920 titles were reviewed and 3,474 were excluded based on title. The remaining 4,446 were reviewed using abstracts and 4,393 were excluded. Fifty-three full text articles were assessed for final eligibility. Fourty-six articles were eliminated because they were reviews, pilot studies, or took place in developed countries. Figure 1 shows the results of the search. Seven eligible studies were identified based upon the eligibility criteria.

Data collected from the eligible articles are shown in Tables 1 and 2. Interventions included a cluster randomized trial, mixed methods study, retrospective comparison of an opt-in text message program, a two-arm proof of concept, single arm trial, a randomized trial, and a single subject design. Five out of seven of the studies showed significant difference between the control and intervention.

**Table 1:** Summary of interventions

Study Author, Year	Participants (Completed)	Sample Of Population	Intervention Duration	mHealth Delivery System	Study Design	Type of Control
Chang, 2012	970	Rural Ugandans; peer health (PH) workers at 10 clinics and 970 patients cared for by the PH workers	26 months	Text message	Cluster randomized trial	Usual care

Lau, 2014	206 (118)	Pregnant women >18 in Cape Town, South Africa	9 months	Text message	Mixed methods study	Usual care

Lepper, 2013	4, 768	Rural and Urban Ugandans	24 months	Text message	Retrospective comparison	Received intervention but no incentives

Meankaew, 2010	534	At risk groups for malaria along the Tha-Myanmar border	12 Months	Text message	Two-arm proof of concept	Usual case follow-up

Odigie, 2011	1176 (1160)	Individuals recruited from the clinics of the Ahmadu Bello University Teaching Hospital in Zaria, Nigeria	24 months	Mobile phone calls	Single arm trial	None

Piette, 2012	200 (181)	Participants living in Honduras and Mexico between 18- 80 years old with high systolic blood pressure (≥140mmHg if nondiabetic; ≥130 mmHg if diabetic) and access to a cell phone or landline	6 weeks	Phone and email	Randomized trial	Usual Care

Tran, 2010	30	Individuals living in Cairo, Egypt with a visible skin lesion	4 weeks	Store and Forward using mobile Phone and internet	Single arm design	None

**Table 2:** Summary of intervention results

Study Author, Year	Intervention Description	Intervention Outcomes	Major Findings	Limitations
Chang, 2012	Peer health workers used mobile phones to call and text senior level providers with patient clinical information and their patients were followed for 26 months. Control patients received usual care.	Health communication	Increased health communication and patient care; median follow-up time for virologic outcomes was 103 weeks per individual; did not demonstrate significant difference between study arms	Phone maintenance; Patient Phone Access; Privacy concerns

Lau, 2014	Individuals were randomly assigned to intervention or usual care group; Intervention group received text messages that contained prenatal health information; baseline knowledge questionnaire was given prior to the intervention and post-intervention. Control patients received usual care.	Responsiveness	No major difference in scores out of the 9 questions asked in intervention and control group; no statistical significant difference between control and intervention	Self-reporting; Loss to follow-up (only 57% completed)

Lepper, 2013	By using Text to Change, which is an opt-in SMS education program, participants were asked questions on various topics with incentives sent to encourage participation; response time, percentage of answered questions, and participation rate. Control patients received intervention but no incentives.	Responsiveness	50% of participants responded within 50 min; In 2009 the median number of questions received was 17; 24 in 2010; 30% of participants never answered any of the quiz questions; in 2009 25% of the questions were answered and 57% in 2010; 79% of the HIV and 78% of the malaria questions were answered, while only 37% were answered for questions regarding to population demographics; Incentives were very effective; Response rates depended on the network provider; the response chance declined with every additional day after sending an incentive via text (Hazard Ratio 0.993, CI 95% 0.981-0.984)	Retrospective setting

Meankaew, 2010	Individuals with malaria were registered onto a system along with the details of their case; as well as a follow-up schedule for them; they were then notified for follow-up using mobile phones and text and graph messages were sent to physicians for analysis. Control patients received usual care follow-up.	Adherence	System followed 534 patients in 2009; Long term follow-up better with system; >90%, self-reported adherence showed high completion rates; the mobile-phone-based case follow-up rates by malaria staff improved significantly	Intervention focused on providers, rather than patients

Odigie, 2011	Oncology patients were given their doctor’s phone number and told to call using their mobile phone regarding their medical care or any questions they needed answered; over 24 months each patient’s phone call and reason for calling was noted in the database with an interview at exit. No control was used.	Adherence	97.6% kept follow-up appointments as opposed to 19.2% who were not in the phone intervention group; patients felt more comfortable having mobile phone access to their doctor; patients preferred mobile phone communication because it helped decrease travel	Some of the patients in the comparison group were recruited through friends, who are referred to as ‘incidental patients’

Piette, 2012	Participants with high BPs received weekly telephone calls from a server in the U.S. using voice over Internet protocol while also being issued a home BP monitor; Patients were reminded to check their BP; Prompts to refill medications, email alerts for health professionals when their patients were having high HP; and the option to sign up a family or friend who would receive a check-up weekly of how they were doing. Control patients received usual care.	Clinical Outcomes	4.2mm Hg decrease in systolic blood pressure with the intervention patients (95% confidence interval- 9.1, 0.7; p=0.09); in the subgroup with high information needs, intervention patients’ average SBPs decreased 8.8mm Hg (-14.2, -3.4, p=0.002); compared with controls interventions patients at follow-up reported fewer depressive symptoms (p=0.004), less medication problems (p<0.0001), better general health (p<0.0001), and greater satisfaction with care (p< 0.004)	Short follow-up period; little to no interaction with patients’ doctors

Tran, 2010	Individuals with visible skin lesions were given a face-to-face consultation with local dermatologists; the dermatologists then used a software-enabled mobile phone to capture images of the skin lesions and then sent the pictures to senior dermatologists for their expertise. No control was used.	Clinical Outcomes	Able to receive expertise advice from specialists; Senior dermatologists were in agreement with diagnoses of on-site junior dermatologist via face-to-face consultation 75% of the time; most typical reasons given by teledermatologist 1 for diagnostic nonagreement were incorrect diagnosis by the on-site physician (3 cases), insufficient history taken (2 cases), and need for an additional test (1 case); for the 2^nd^ teledermatologist, most common reasons for diagnostic nonagreement was insufficient history taken (3 cases), incorrect diagnosis by the on-site physician (2 cases), need for additional test (2 cases), and poor image quality (1 case)	Face-to-face consultations and mobile phone examination were not completed by dermatologists at the same level of training

### 3.2 Study Characteristics & Results of Individual Studies

Tables 1 and 2 provide a summary of the seven studies that met eligibility criteria, which were diverse in regards to sample size, sample population, intervention duration, mHealth delivery system, study design, and type of control. Sample sizes ranged from 30 to 4,768 participants and the intervention duration ranged from 4 weeks to 26 months.

Study design included two randomized trials (Chang et al., 2012; Piette et al., 2012), one mixed methods study (Lau et al., 2014), one two-arm proof of concept (Meankaew et al., 2010), two single arm trials (Odigie et al., 2012; Tran et al., 2011), and one retrospective comparison (de Lepper et al., 2013). Three of the studies used a usual care group (Lau et al, 2014; Piette et al, 2012; Chang et al., 2012), two studies had no control (Odigie et al., 2012, Tran et al., 2011), one study used a usual case follow-up (Meankaew et al., 2010), and one study received the intervention but had no incentives (de Lepper et al., 2013).

Table 2 illustrates a summary of the intervention results of the studies that met the inclusion criteria. Two of the studies measured responsiveness (Lau et al., 2014; de Lepper et al., 2013), two measured adherence (Meankaew et al., 2010; Odigie et al., 2012), two measured clinical outcomes (Piette et al., 2012; Tran et al., 2011), and one measured health communication (Chang et al., 2012).

Two of the studies did not demonstrate significant difference between the control and intervention group (Chang et al., 2012; Lau et al., 2014). One of the studies had no statistical significant difference (all p>0.05) between control and intervention and had no major difference in scores out of the 9 questions that were asked in each group pertaining to prenatal health information. There was a significant loss to follow-up during the study with only 57% of participants retained at exit (Lau et al., 2014). Although the intervention failed to improve prenatal and antenatal health information, evidence from self-reported behavior and the focus groups show text messages have the potential to inspire change in health-seeking behavior (Lau et al., 2014) No statistical significance was found between study arms when peer health workers used mobile phones to call and text senior-level providers with patient clinical information; although it did increase health communication and patient care (Chang et al., 2012).

Mobile phone-based case follow-up rates by malaria staff improved significantly when individuals were registered onto a system along with details of their case; text and graph messages were sent to physicians for analysis (Meankaew et al., 2010) 97.6% of patients kept follow-up appointments as opposed to the 19.2% who were not in the intervention group; the intervention group consisted of oncology patients having their primary care doctor’s mobile phone numbers in order to ask any medical questions (Odigie et al., 2012). Patients preferred mobile phone communication because it decreased travel (cost-effective) (Odigie et al., 2012). A significant decrease in systolic blood pressure (SBP) was shown with intervention patients in a study of individuals with high blood pressure (Piette et al., 2012). A 4.2 mm HG decrease in systolic blood pressure with a 95% confidence interval was found. In the subgroup with high information needs, intervention patients average SBPs decreased 8.8 mmHg (-14.2, -3.4, p=0.002); intervention patients at follow-up reported fewer depressive symptoms (p=0.004), less medication problems (p<0.0001), better general health (p<0.0001), and greater satisfaction with care (p<0.004) (Piette et al., 2012). Finally, in a study using dermatologists, senior dermatologists were in agreement with diagnoses of on-site junior dermatologists via face-to-face consultation 75% of the time (Tran et al., 2011).

## 4. Discussion

This systematic review identified mHealth interventions completed in low and middle income countries measuring patient outcomes. After reviewing 7,845 articles resulting from search terms, only seven articles met the eligibility criteria to be included in this review. Currently there is little evidence on mHealth interventions in developing countries, and existing studies are very diverse; however initial studies show changes in clinical outcomes, adherence, and health communication. In addition, articles reviewed noted an ability to receive expert advice, and offered a wide range of benefits using mHealth. Based on the articles reviewed, mHealth provided new ways to interact with the provider and offered new forms of cost-effective education. Finally, participants tended to be more responsive when technology was involved and the use of mHealth decreased travel time.

Based on this review, while there is little evidence in the literature, studies suggest mHealth offers improved communication with providers, decrease in travel time, ability to receive expert advice, changes in clinical outcomes, and new forms of cost-effective education. For example, individuals with high blood pressure living in Honduras and Mexico had their systolic blood pressure decrease 4.2 mmHg after receiving telephone calls from a server in the United States using voice-over Internet protocol and a home blood pressure monitor (Piette et al., 2012). Participants received email alerts to refill medications and alerts were sent to their physician when their patient’s BP was too high (Piette et al., 2012). In another study, individuals living in Cairo, Egypt with a visible skin lesion were able to have a face-to-face consultation with an on-site dermatologist, who then could capture images of the skin lesions to send to senior dermatologists for their expertise (Tran et al., 2011). As a result, the participants were able to decrease travel time, saving on travel expenses (Tran et al., 2011). Finally, individuals living with malaria in the Tha-Myanmar border; were registered onto a system that allowed them to receive follow-up using mobile phones. Text and graph message were sent to physicians for analysis. Long-term follow up was better with the system; self-reported adherence showed high completion rates; and the mobile-phone-based case follow-up rates by malaria staff improved significantly (Meankaew et al., 2010).

Many developing countries are faced with a scarcity of resources, both human and technological, and there are many barriers to patient access to care and health knowledge (Littman-Quinn et al., 2013). Other obstacles developing countries face include malfunctioning mobile devices, unreliable IT infrastructure, cultural misalignment between IT and healthcare providers, and electricity problems (Littman-Quinn et al., 2013). In order to substantiate public investment, more evidence of how mHealth can successfully improve healthcare delivery for resource-poor countries needs to be completed (Chib, 2013). Based on this review, it stands to reason if mHealth is available in developing countries, it could improve education and training of healthcare workers, provide a wider range of health communication with providers, and be more cost-effective (Chib, 2013). However, more evidence is needed to support and direct system-level resource investment.

A significant finding of this review is the fact that few studies have been conducted using mHealth in low and middle income countries, while measuring outcomes. In addition, interventions are not clearly described limiting replication. Future studies are needed to provide evidence on the effectiveness and implementation of mHealth. Based on this review, mHealth could provide ways to address chronic diseases in order to increase health knowledge in developing countries. mHealth has increased health communication between the patient and their provider offering an easier method to discuss medical issues. However, it will be necessary for future studies to be methodologically sound and describe intervention components clearly in order to build the evidence base for mHealth and guide implementation in developing countries.

Our findings match other reviews conducted to determine the effectiveness of mHealth strategies. Specifically, reviews recently conducted on mHealth strategies for frontline health workers, SMS text messaging to promote medication adherence, mHealth usage to improve antenatal/postnatal care and immunization, mHealth behavior change interventions, and mHealth interventions for maternal and child health agree that few studies exist, however evidence is promising (Lee et al., 2015; Agarwal et al., 2015; DeKoekkoek et al., 2015; Watterson et al., 2015; Pfaeffli et al., 2015). In addition, our findings agree with recently published reviews that interventions are often ambiguous and of poor methodological quality (Lee et al., 2015; Pfaeffli et al., 2015) Reviews suggest mHealth may be effective in changing behaviors, specifically through simple text messaging, however, rigorous trials are needed (Pfaeffli et al., 2015; Watterson et al., 2015; DeKoekkoek et al., 2015). Finally, it is recommended to include more evaluation of impacts and patient outcomes, in addition to the more common process measures (Lee et al., 2015).

There are several limitations worth addressing. First, the search criteria was limited to articles published in English between 2000 and 2014. Second, the review was limited to completion of an intervention in a low or middle income country, measurement of patient outcomes, and participants 18 years of age or older. In addition, there is a possibility that more mobile health technology interventions have been completed, but have not yet been published. As a result of the limited information available, conclusions from this review are qualitative and will hopefully guide future research with mobile health technology involved in order to find more valuable uses of mHealth.

## 5. Conclusions

Currently there is not much evidence on mHealth interventions in developing countries, and existing studies are very diverse, but initial studies suggest mHealth offers a wide range of benefits, including improvement in clinical outcomes, adherence, health communication, and ability to receive expert advice. Based on the articles reviewed, mHealth provided new ways to interact with the provider and sparked new forms of cost-effective education. In addition, participants tended to be more responsive when technology was involved and use of mHealth decreased travel time. These results agree with other reviews conducted recently on use of mHealth for specific outcomes. Based on our review, future research should measure effectiveness and implementation of mobile health technology, specifically in the context of patient outcomes. Studies should also investigate strategies to provide access to mHealth into developing countries.
